# Response to comments by Kong et al.

**DOI:** 10.1016/j.mocell.2025.100288

**Published:** 2025-10-17

**Authors:** Hoyun Kwak, Wijin Jeon, Nui Ha, Soon-Gu Kwon, Jae Young Seong

**Affiliations:** 1 Neuracle Science Co, Ltd, Seoul 02841, Republic of Korea; 2 Graduate School of Biomedical Sciences, Korea University College of Medicine, Seoul 02841, Republic of Korea

We appreciate the opportunity to address the commentary provided by Kong et al. regarding our recent publication, “Is FAM19A5 an adipokine? Peripheral FAM19A5 in wild-type, FAM19A5 knockout, and LacZ knockin mice.” This letter highlights several important critiques concerning our conclusions on the peripheral expression of FAM19A5, specifically (1) the interpretation of *FAM19A5* mRNA expression data in adipose tissue, (2) the validity and design of our ELISA method for detecting FAM19A5, and (3) the exclusion of S1PR2 as a functional receptor for FAM19A5. We value this opportunity for scientific exchange and welcome the continued discussion of the physiological role of FAM19A5. We address each of these points, providing clarification and supporting evidence on the basis of our experimental data and published literature.

## DISCREPANCY BETWEEN FAM19A5 ISOFORM 2 mRNA LEVELS AND X-GAL STAINING IN THE ADIPOSE TISSUE

Kong et al. noted an inconsistency between the relatively high expression level of *FAM19A5* isoform 2 mRNA in adipose tissue and the fact that X-gal staining was not detectable in the same tissue, whereas weak signals were observed in other peripheral organs, such as the heart, small intestine, and testis. However, notably, the *LacZ* reporter system used in our *FAM19A5-LacZ* knock-in mice reflects the combined transcriptional activity of both isoform 1 and isoform 2, not isoform 2 alone (Shahapal et al., 2019, Shahapal et al., 2025). Therefore, directly comparing isoform 2 mRNA levels with LacZ reporter activity may not be appropriate; rather, comparisons should be made with the total expression of *FAM19A5* transcripts. We acknowledge that total *FAM19A5* mRNA levels in adipose tissue are similar to or greater than those in the heart, small intestine, and testis. However, as described in our results, the X-gal signals observed in the small intestine and testis are extremely weak and restricted to limited cell populations and, in some cases, may reflect nonspecific signals or endogenous β-galactosidase activity (Kwak et al., 2024). The mRNA levels detected in these tissues were also low, which may account for the marginal reporter expression observed. Furthermore, differences in enzymatic background and cell type specificity can contribute to variability in X-gal staining intensity across organs.

## EVALUATION OF FAM19A5 ELISA METHODOLOGIES

The authors questioned the validity of our binding protein-based ELISA using the LRRC4B fragment, arguing that it does not follow the standard ELISA protocol. However, similar or slightly modified ELISA formats employing high-affinity binding reagents instead of traditional capture antibodies have been reported in the literature for target-specific protein quantification (Heyduk et al., 2018, Lee and Zeng, 2017, Shang et al., 2018).

**Fig. 1:**
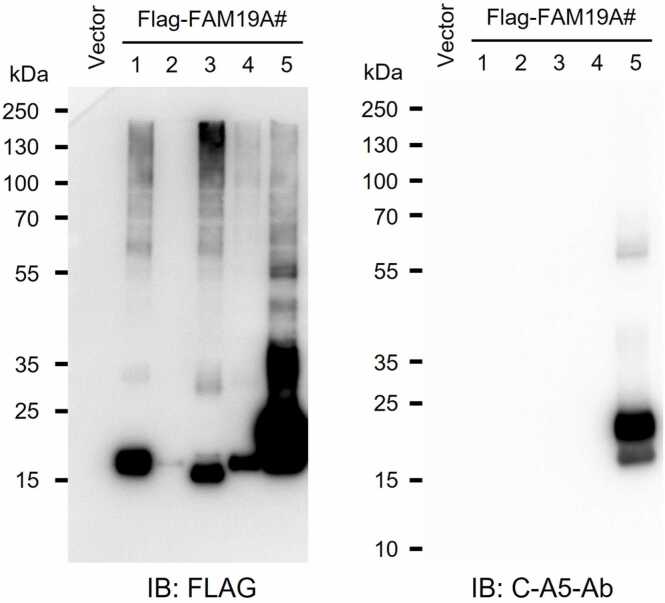
Western blot analysis of the human FAM19A family (FAM19A1-A5). Human *FAM19A1-A5* coding sequences (signal peptide-containing isoforms) were cloned into a pcDNA3.1 vector with a C-terminal FLAG tag. HEK293 cells were transiently transfected and cultured for 48 hours, after which cell lysates were collected. Samples were resolved under reducing conditions on 10% to 20% SDS-PAGE, transferred to PVDF membranes, blocked, and probed with anti-FLAG antibody to detect all FLAG-tagged family members (left) or with C-A5-Ab to specifically detect FAM19A5 (right). The calculated mature (postsignal-peptide, unglycosylated) molecular weights are approximately: FAM19A1, 10.2 kDa; FAM19A2, 10.0 kDa; FAM19A3, 9.9 kDa; FAM19A4, 10.1 kDa; and FAM19A5, 9.9 kDa. The empty-vector (Vector) lane serves as a negative control. The C-A5-Ab signal was detected exclusively in the FAM19A5 lane, confirming the antibody’s specificity under these conditions.

The LRRC4B(453-576) fragment, which we used as the capture protein in our ELISA, was previously characterized to bind FAM19A5 with high affinity, with an equilibrium dissociation constant (K_D_) of 32 pM, as determined by surface plasmon resonance analysis (Kim et al., 2025). In our assay, this fragment was immobilized on an ELISA plate to capture FAM19A5 from test samples. Detection was then performed via the use of a deimmunized monoclonal antibody (C-A5-Ab) that specifically recognizes the C-terminal region of human FAM19A5 (Kim et al., 2025). This antibody binds to a conformational epitope in the C-terminal domain and was validated to selectively detect FAM19A5 via Western blot analysis (Fig. 1). Notably, the ELISA method we developed is highly sensitive and capable of detecting FAM19A5 at concentrations as low as several tens of picograms per milliliter.

Importantly, this ELISA platform has been validated using samples from *FAM19A5* knockout mice, confirming the absence of nonspecific reactions in CSF and serum/plasma samples from mice, rats, monkeys, and humans. Moreover, the ELISA platform is currently being employed in an ongoing clinical trial for pharmacodynamic analysis (Kim et al., 2025). The assay was further qualified by a central laboratory under regulatory standards, confirming its analytical specificity, sensitivity, and suitability for use in human biofluid studies (ClinicalTrials.gov registration: NCT05143463).

## FAM19A5 AS AN ADIPOKINE

Publications from authors and other research groups have proposed that FAM19A5 is highly expressed in adipose tissue and functions as an adipokine (Huang et al., 2021, Wang et al., 2018). Using a human cDNA library, the authors reported elevated *FAM19A5* transcript levels in adipose tissue via TaqMan real-time PCR analysis. They further supported the assertion that FAM19A5 is highly expressed in adipose tissue by performing immunohistochemistry, ELISA, and cytometric bead assay, which use a combination of an in-house polyclonal anti-rabbit FAM19A5 antibody and commercially available antibodies, and reported elevated FAM19A5 levels in adipose tissue.

However, these results differ from both our findings and publicly available transcriptomic datasets (Karlsson et al., 2021, Kwak et al., 2024, Mathys et al., 2019, Sjöstedt et al., 2020, Zeisel et al., 2018). Using our previously described high-sensitivity ELISA platform and Western blot analysis, we attempted to detect the FAM19A5 protein in adipose tissue at the protein level (Kwak et al., 2024). In all cases, the results fell below the detection limit, indicating that FAM19A5 is either absent or present at extremely low concentrations in adipose tissue.

If FAM19A5 was expressed in adipose tissue at levels comparable to those in the brain, it would be readily detectable by direct protein detection assays such as Western blotting and ELISA. The failure to detect FAM19A5 in adipose tissue via these standard biochemical assays suggests that previously reported protein-level findings may warrant careful re-evaluation.

## SINGLE-NUCLEI RNA SEQUENCING ANALYSIS OF HUMAN ADIPOSE TISSUE

**Fig. 2:**
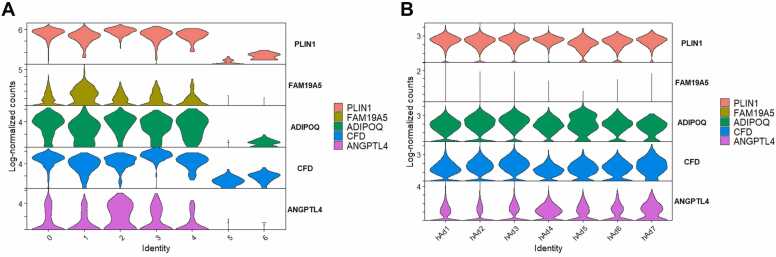
RNA expression of *FAM19A5* and canonical adipokine genes in *PLIN1*-positive human adipocytes. (A) Single-nuclei RNA-seq data from the GSM5699630 sample of GSE189346 (Whytock et al., 2022; https://www.ncbi.nlm.nih.gov/geo/query/acc.cgi?acc=GSM5699630) were reanalyzed. “Subject A_Adipocytes” and “Subject B_Adipocytes” were merged and processed in Seurat v5.3.0. Cells with <200 detected genes, <10,000 UMIs, or >28% mitochondrial reads were excluded; data were log-normalized (NormalizeData), highly variable genes identified (FindVariableFeatures), scaled, and reduced by PCA (top 30 PCs) followed by UMAP, with clustering at a resolution of 0.5 (FindClusters). Violin plots (VlnPlot) show log-normalized counts for *FAM19A5*, *PLIN1*, *ADIPOQ*, *CFD*, and *ANGPTL4* within *PLIN1*-positive adipocyte clusters. *FAM19A5* expression levels were markedly lower than those of canonical adipokine genes, averaging 70-, 20-, 40-, and 6-fold lower than *PLIN1*, *ADIPOQ*, *CFD*, and *ANGPTL4*, respectively, indicating that *FAM19A5* is expressed at relatively low levels compared with established adipokines in this dataset. (B) Preprocessed and clustered human adipocyte single-cell data (human_adipocytes.rds) were obtained from the White Adipose Atlas (Emont et al., 2022; https://gitlab.com/rosen-lab/white-adipose-atlas) and visualized in Seurat v5.3.0 using VlnPlot with the data slot to maintain the original normalization. Across adipocyte subtypes (hAd1-hAd7), *FAM19A5* signals were nearly absent compared with canonical adipokines; mean *FAM19A5* levels were 550-, 360-, 200-, and 130-fold lower than *PLIN1*, *ADIPOQ*, *CFD*, and *ANGPTL4*, respectively. Notably, *FAM19A5* expression levels observed in this dataset were substantially lower than those in the Whytock et al. dataset, further supporting negligible *FAM19A5* expression in *PLIN1*-positive human adipocytes in an independent cohort.

The authors reanalyzed single-nuclei RNA sequencing data from human adipose tissue and noted that *FAM19A5* transcripts were detected in clusters expressing the adipocyte marker *PLIN1* (Whytock et al., 2022). To further evaluate the relative abundance of *FAM19A5*, we performed an additional analysis of the same dataset and compared its expression levels in *PLIN1*-positive adipocytes with those of established adipokine genes, including *ADIPOQ*, *CFD*, and *ANGPTL4*. This analysis revealed that *FAM19A5* transcript levels were consistently lower than those of these canonical adipokines, being, on average, approximately 70-, 20-, 40-, and 6-fold lower than those of *PLIN1*, *ADIPOQ*, *CFD*, and *ANGPTL4*, respectively (Fig. 2A). In addition, other single-cell RNA sequencing datasets of human adipose tissue (Emont et al., 2022) revealed negligible levels of *FAM19A5* transcripts compared with those of *PLIN1, ADIPOQ*, *CFD*, and *ANGPTL4*, which were approximately 550-, 360-, 200-, and 130-fold lower, respectively (Fig. 2B).

## FAM19A5 PROTEIN USED FOR S1PR2 BINDING

The authors raised questions about the nature of the FAM19A5 protein used in our S1PR2-binding assays. The FAM19A5 protein employed in our experiments corresponded to the mature, secreted form lacking the native N-terminal signal peptide (Kim et al., 2025, Kwak et al., 2024). This finding was confirmed through N-terminal sequencing and is consistent with the mature form described in Wang et al. On Western blot analysis, HEK293-derived nonglycosylated mature FAM19A5 migrated at ∼12 kDa, despite the calculated 9.9 kDa for the mature polypeptide after signal peptide removal. This modest upward shift is consistent with the known tendency of small secreted and cysteine-rich proteins to migrate anomalously in SDS-PAGE (Rath et al., 2009, Shi et al., 2012, Tiwari et al., 2019). Although potential phosphorylation at Thr65 has been predicted in post-translational modification database (PhosphoSitePlus), to our knowledge, this modification has not been experimentally verified. More importantly, because our recombinant FAM19A5 migrates to the same position as the endogenously expressed FAM19A5 from mouse brain on electrophoresis, it can be considered to closely resemble the natural form of FAM19A5.

For efficient and scalable expression, we designed a construct encoding the human IgG kappa chain signal peptide, a hexa-histidine tag, and a TEV protease cleavage site upstream of the coding region for *FAM19A5* isoform 1, excluding its endogenous signal peptide. The recombinant protein was expressed in Expi293 cells, purified via Ni-NTA affinity chromatography, and treated with TEV protease to remove the His-tag and cleavage site. The final preparation—which was tag-free and expressed in a eukaryotic system—was used for all in vitro functional studies related to the S1PR2 interaction.

While Wang et al. also evaluated the interaction between FAM19A5 and S1PR2 via purified protein, their reported findings differ from ours. Importantly, it remains unclear whether the FAM19A5 protein is expressed in a eukaryotic or prokaryotic system or whether consistent results are obtained across different expression platforms. Clarification of these methodological details would be helpful for interpreting the discrepancies between studies and for determining whether the observed differences stem from variations in protein folding, post-translational modifications, or assay conditions.

In conclusion, our data suggest that FAM19A5 may not currently meet the criteria for classification as an adipokine. Instead, multiple lines of evidence indicate that FAM19A5 is predominantly expressed in the brain, with minimal or undetectable levels in adipose tissue under our experimental conditions. Our validated assays and recombinant protein studies did not provide evidence of interactions with S1PR2. Nevertheless, we acknowledge that this approach may be subject to certain constraints under specific conditions and that methodological differences across studies could plausibly influence the observed outcomes. We propose that differences in antibody specificity, protein preparation, and assay format likely contribute to the observed discrepancies. Continued investigations of FAM19A5 biology, supported by rigorous validation and transparent reporting, will be essential for integrating diverse findings into a more comprehensive understanding.

## AUTHOR CONTRIBUTIONS

**Wijin Jeon:** Data curation. **Hoyun Kwak:** Writing – review & editing, Writing – original draft. **Soon-Gu Kwon:** Writing – review & editing. **Nui Ha:** Data curation. **Jae Young Seong:** Writing – review & editing, Writing – original draft, Supervision.

## DECLARATION OF COMPETING INTERESTS

Hoyun Kwak, Wijin Jeon, Nui Ha, Soon-Gu Kwon, and Jae Young Seong are employed by Neuracle Science Co, Ltd. Soon-Gu Kwon and Jae Young Seong are shareholders of Neuracle Science Co, Ltd.
